# Overprinting of TPU onto PA6 Substrates: The Influences of the Interfacial Area, Surface Roughness and Processing Parameters on the Adhesion between Components

**DOI:** 10.3390/polym16050650

**Published:** 2024-02-28

**Authors:** Janez Slapnik, Rebeka Lorber, Irena Pulko, Miroslav Huskić, Klementina Pušnik Črešnar

**Affiliations:** Faculty of Polymer Technology, Ozare 19, 2380 Slovenj Gradec, Slovenia; janez.slapnik@ftpo.eu (J.S.); rebeka.lorber@ftpo.eu (R.L.); irena.pulko@ftpo.eu (I.P.); miroslav.huskic@ftpo.eu (M.H.)

**Keywords:** injection moulding, additive manufacturing, fused filament fabrication, overprinting, adhesion

## Abstract

The hybridisation of injection moulding (IM) and additive manufacturing (AM) offers the opportunity to combine the high productivity of IM and the high flexibility of AM into a single process. IM parts can be overprinted through fused filament fabrication (FFF) to allow for the customisation of parts or to add new functionalities. However, the right material pair must be chosen, and processing parameters must be optimised to achieve suitable adhesion between the components. The present study dealt with the investigation of the influence of the interfacial area, substrate surface roughness and overprinting processing parameters on the adhesion between the polyamide 6 (PA6) substrate and thermoplastic polyurethane (TPU) rib overprinted via FFF. PA6 substrates were produced through the IM of plates into a mould with different textures to obtain substrates with three different surface roughnesses. The ribs with varied interfacial areas were overprinted onto produced substrates using a desktop FFF 3D printer. To study the effect of overprinting processing parameters, the ribs were overprinted under varying printing and substrate temperatures and printing speeds according to the Box–Behnken design of experiments (DoE). The chemical composition and thermal properties of used materials were determined via attenuated total reflectance–Fourier transform infrared spectroscopy (ATR-FTIR), thermogravimetric analysis (TGA), differential scanning calorimetry (DSC) and dynamic mechanical analysis (DMA). The surface properties of prepared substrates were studied via digital optical microscopy (OM), through surface roughness measurements using a confocal microscope, through contact angle (CA) measurements and through the determination of free surface energy (SFE). The adhesion between the components was determined by evaluating the tear-off strength using a universal testing machine (UTM). With an increasing interfacial area, the tear-off strength decreased, while substrate surface roughness had no statistically significant effect. Overprinting parameters influenced the tear-off strength in the order of printing speed > printing temperature > substrate temperature. High values of tear-off strength were found for the lowest printing speed, while there were no important differences found between the middle and upper values. With increasing printing and substrate temperatures, the tear-off strength increased linearly. The highest value of tear-off strength (0.84 MPa) was observed at a printing temperature, substrate temperature and printing speed of 250 °C, 80 °C and 2 mm/s, respectively.

## 1. Introduction

With further technological development, plastic products are becoming increasingly more complex and often require the fulfilment of several functionalities within a single part. IM is one of the most widespread technologies in industry for producing plastic parts with complex geometries with high productivity. In a typical IM process, a plastic granulate is melted and homogenised in a plasticising unit and injected into a mould cavity where it is solidified to form a plastic part [1]. To produce more complex parts, a multi-material IM process is often employed in which at least two plasticising units are integrated into a single machine to enable the injection of multiple materials into a single mould. This process enables increased design flexibility but still has limitations in terms of the geometries that are possible to produce [2]. Moreover, each geometric part requires the production of a specific mould, which is lengthy and expensive, making IM inherently inflexible technology in terms of the production of custom parts [1,2]. On the other hand, AM has complete design flexibility as it enables the production of almost any geometry directly from a computer-aided design (CAD) model, but it has much lower productivity [3,4]. The hybridisation of IM and additive manufacturing offers the opportunity to combine the high productivity of an IM process and the design flexibility of AM. One way of hybridising these technologies is to mould the plastic part and subsequently overprint additional structures onto it via material extrusion AM, such as FFF. In this way, injection moulded parts can be customised, or new functionalities can be achieved by adding structures that are not possible to produce via IM [5,6]. In multi-material IM, the materials of different chemical compositions are combined into a part [2,7]. Achieving good adhesion between individual components is of paramount importance and is especially critical when combining chemically dissimilar materials, as in our study. Here, an understanding of the adhesion phenomena, which includes surface chemistry, physics, rheology, polymer chemistry, stress analysis, polymer physics and fracture analysis is of growing importance. In general, adhesion is known as the interatomic and intermolecular interactions at the interface of two surfaces. Due to the complexity of adhesion phenomena, defining the mechanism of adhesion in simple terms is difficult. Therefore, the three major adhesion mechanisms are defined to understand polymer adhesion and to clarify how the interfaces are actually adhering. Thus, mechanical coupling, molecular bonding and thermodynamic adhesion are considered [8,9,10,11,12]. Which material pairs adhere well to each other and how processing parameters influence the adhesion between components is relatively well studied and understood for a multi-material IM process [2,7]. In contrast, only a few studies have dealt with the investigation of the influence of processing parameters on the adhesion of overprinted injection moulded parts and most of these studies focused on combining materials with the same chemistry [5,6]. 

This study aimed to determine how various factors influence the adhesion between an injection moulded substrate from PA6 and an overprinted structure from TPU. This material combination was chosen as it is attractive due to the possibility of achieving different mechanical properties within a single part. In detail, this study investigated the influences of the substrate surface roughness, the overprinted part’s interfacial area and overprinting processing parameters (printing and substrate temperatures and printing speed) on the tear-off strength between the substrate plate and overprinted rib. It was hypothesised that increased surface roughness should lead to increased adhesion due to the higher specific surface area and mechanical interlocking, while the interfacial area should also importantly influence the tear-off strength due to different contributions of the notch effect and shrinkage-related residual stresses in the overprinted structure. In addition, all three investigated processing parameters should importantly influence the adhesion by controlling the heat transfer at the interface, with higher temperatures resulting in a better welding of the components but also in possible detrimental effects due to degradation.

## 2. Materials and Methods

### 2.1. Materials

PA6 Akulon^®^ K222-D (DSM Engineering Materials, Emmen, The Netherlands) was used for the preparation of specimens for material characterisation and substrates. According to the manufacturer, the PA6 had a melt volume flow rate (MVR) of 185 cm^3^/10 min (ISO 1133 [13], 275 °C, 5 kg), a melting point of 220 °C (ISO 11357-3 [14], 10 K/min) and a density of 1.13 g/cm^3^ (ISO 1183 [15]). TPU filament (Plastika Trček, Ljubljana, Slovenia) with a ShoreA hardness of 89A was used for the preparation of specimens for the material characterisation and overprinting of ribs onto substrates.

### 2.2. Sample Preparation

#### 2.2.1. Injection Moulding of Material Characterisation Test Specimens

The PA6 was used as supplied. The TPU filament was granulated using a Scheer SGS 25-E4 (Maag Pump Systems AG, Oberglatt, Switzerland) and dried prior to processing in a DryWell (Vismec Srl, Camposampiero, Italy). The moisture content of the materials was determined using an HX 204 (Mettler-Toledo GmbH, Greifensee, Switzerland) moisture analyser, and it was below 0.2 wt.%. Test specimens for material characterisation were produced according to ISO 527-2 [16] (type 1BA) and ISO 179-1 [17]. Test substrates were produced in a mould based on the geometry for the production of test specimens for the determination of moulding shrinkage according to the ISO 294-4 standard [18] (dimensions of 60 mm × 60 mm × 4 mm). The mould had interchangeable inserts (Richard Hiebler GmbH, Stainz, Austria) with different textures with target line roughness values *R*_a_ of 0.2 µm, 0.8 µm and 3.2 µm, designated by the VDI 3400 standard [19] as numbers 6, 18 and 30, respectively. All samples were injection moulded using a CX 50-180 (KraussMaffei Technologies GmbH, München, Germany) injection moulding machine with a screw diameter of 30 mm, a length to diameter (L:D) ratio of 23.3:1 and a maximum clamping force of 500 kN. The injection moulding processing parameters are summarised in Table 1.

#### 2.2.2. Overprinting of TPU onto Substrates

**Figure 1:**
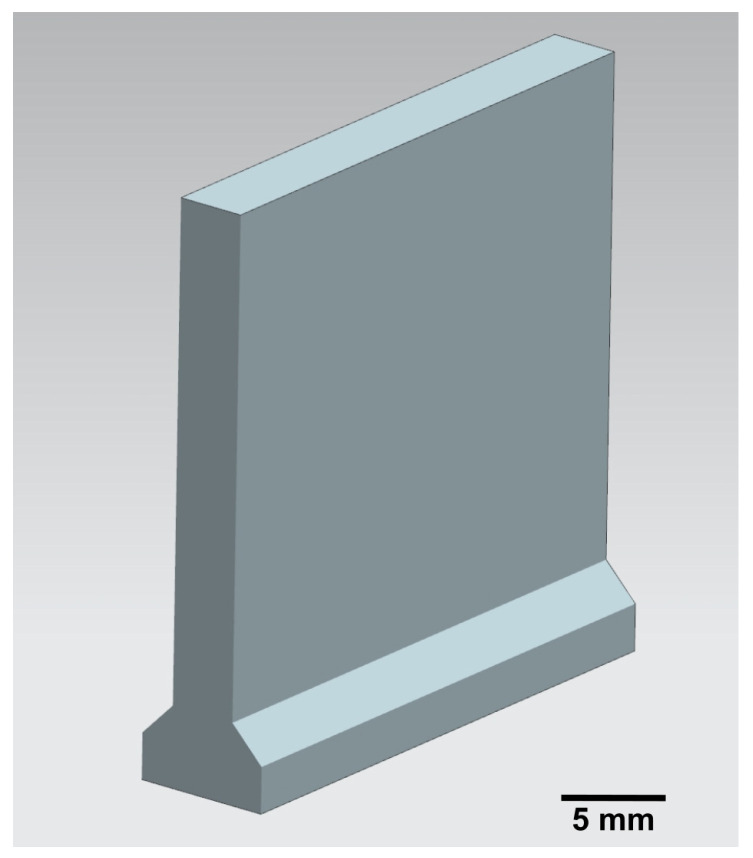
Isometric projection of the CAD model of a rib with an interfacial thickness of 6 mm.

The rib structures for the testing of the tear strength were overprinted onto injection moulded PA6 substrates using an Ender-3 V2 Neo (Creality, Shenzen, China) desktop FFF 3D printer. Three different geometries of ribs with a height of 25 mm, width of 25 mm, clamping thickness of 3 mm and varied interfacial thicknesses (3 mm, 6 mm and 9 mm) were overprinted, with the geometry of a rib with an interfacial thickness presented in Figure 1.

The substrates were fixed onto the build platform using a double-sided adhesion tape. The levelling of the nozzle and the substrate was performed by setting the gap on four calibration points located on the edges of the substrates using 0.1 mm thick printing paper and applying the required Z-axis offset. The G-code was prepared using a Cura 5.3.0 (Ultimaker, Utrecht, The Netherlands) slicer. The bed temperature was set to achieve the desired temperature (±1 °C) of the substrate (*T*_sub_) measured using a RAY 31.1136 (TFA Dostmann GmbH & Co. KG, Wertheim am Main, Germany) infrared thermometer. Samples that varied in substrate surface roughness and interfacial area thickness were designated according to the roughness (R6, R18, and R30) and thickness (T3, T6, and T9) values and were prepared using the processing parameters summarised in Table 2.

The influences of three factors (*T*_print_, *T*_sub_, and *V*_print_) on rib tear strength were investigated using a Box–Behnken design of experiments with full randomisation and one central point. The ribs were overprinted onto substrates with an R30 surface roughness designation. Minitab 19.2 (Minitab Inc., State College, PA, USA) statistical software was used for designing the experiment and statistical analysis. The experimental boundary conditions of the investigated factors were determined empirically, and the constant parameters were the same for all experiments. The experimental design is presented in Table 3.

### 2.3. Characterisation

#### 2.3.1. Attenuated Total Reflectance–Fourier Transform infrared Spectroscopy (ATR-FTIR)

ATR-FTIR spectra were measured on a Spectrum 65 (Perkin Elmer, Waltham, MA, USA) spectrometer in the wavenumber range from 4000 cm^–1^ to 600 cm^–1^, with a resolution of 4 cm^−1^ at room temperature (RT). Each spectrum was determined as an average of 32 scans. All spectra presented here were baseline corrected and smoothed after the measurement.

#### 2.3.2. Thermogravimetric Analysis

The decomposition behaviours of the injection moulded test specimens were investigated using a TGA/DSC 3+ (Mettler-Toledo GmbH, Greifensee, Switzerland) simultaneous TGA/DSC instrument in 40 µL aluminium crucibles. Non-isothermal measurements were performed by heating the samples from 40 °C to 550 °C, with a heating rate of 10 K/min in a nitrogen (N_2_) atmosphere (20 mL/min). Isothermal measurements were performed by heating the samples from 40 °C to the defined temperature with a heating rate of 50 K/min in the N_2_ atmosphere (20 mL/min), followed by a 20 min isothermal segment in a N_2_ or oxygen (O_2_) atmosphere (20 mL/min).

#### 2.3.3. Differential Scanning Calorimetry

Thermal properties were determined using a DSC 2 (Mettler-Toledo GmbH, Greifensee, Switzerland) calorimeter in 40 µL aluminium crucibles. Specimens were prepared from tensile test specimens. The samples were tested in the N_2_ atmosphere (20 mL/min), with a temperature range of 25 °C to 240 °C and −70 °C to 240 °C for the PA6 and TPU, respectively. The heating and cooling rates were 10 K/min, and the isothermal segments were set to 5 min and 1 min before the heating and cooling runs, respectively. The degree of crystallinity (*X*_c_) of the PA6 was calculated according to the following equation:

(1) $$X_{c}=\frac{\Delta H_{m}}{\;{∆H}_{0}}\times 100\%$$

where Δ*H*_m_ is the sample melting enthalpy, and Δ*H*_0_ is the melting enthalpy of 100% crystalline PA6 (230 J/g) [20].

#### 2.3.4. Optical Microscopy

Optical micrographs were captured using a VHX-7000 (Keyence, Osaka, Japan) digital microscope using a VH-ZST lens, captured using reflective illumination at 20× and 200× magnification for the overprinted ribs and surfaces of substrates, respectively. 

#### 2.3.5. Surface Roughness Measurements

The line roughness (*R*_a_) and the surface roughness (*S*_a_) were determined with a confocal microscope Microprof MPR1080 (Fries Research and Technology (FRT) GmbH., Bergisch Gladbach, Germany). Line roughness was determined by averaging the values of 5 randomly placed parallel data lines along the measured surface. Surface roughness was determined on a 2 mm × 2 mm square area with a cut-off wavelength (*λ*c) of 0.885 to distinguish between the roughness and waviness of samples.

#### 2.3.6. Contact Angle Measurements with Free Surface Energy Calculation

To highlight the phenomena of the surface properties of TPU and PA6 on the further adhesion, the injection moulded test specimens were investigated with contact angle (CA) measurements and surface free energy (SFE) calculations [21]. The CAs of PA and TPU were measured using a goniometer from DataPhysics (Filderstadt, Germany) and were performed with two different liquids: ultra-pure water (Millipore, Burlington, MA, USA) and diiodomethane with a purity of 99% (Sigma-Aldrich, Burlington, MA, USA). The droplet volume was 3 µL, and results were obtained by averaging the values obtained from at least three liquid droplets per surface. In this way, static contact angles (SCAs) were measured at room temperature. The approach of Owens, Wendt, Rabel and Kaelble (OWRK) represents one of the most common methods for calculating the SFE of polymeric materials, using water and diiodomethane as measuring fluids [22]. The dispersive and polar phases of the surface energy of the TPU and PA were calculated by using two liquids with known surface tensions (diiodomethane with a surface tension of 50.8 mN/m, polar component of 2.3 mN/m and dispersive component of 48.5 mN/m; and water with a surface tension of 72.8 mN/m, polar component of 51.0 mN/m and dispersive component of 21.8 mN/m).

#### 2.3.7. Tear-Off Strength Tests

Tear-off strength was determined using an Ag-X plus 10 kN (Shimadzu, Kyoto, Japan) universal testing machine with a special adapter for the measurement of tear-off strength [5]. The preload was 10 N, and the testing speed was 1 mm/min. The tear-off strength was calculated based on the measured interfacial area between the rib and the substrate and the force required for the tear-off. The presented results are the average values of five measurements.

## 3. Results and Discussion

### 3.1. Material Characterisation

#### 3.1.1. Chemical Composition

**Figure 2:**
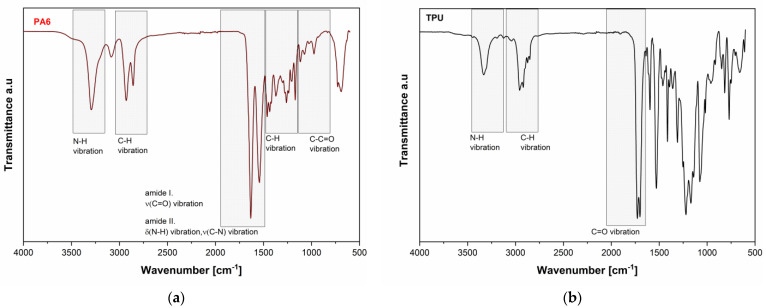
A comparison of ATR-FTIR spectra in the range from 4000 cm^−1^ to 600 cm^−1^ of (**a**) TPU; (**b**) PA6.

The spectral sensitivity and depth analysis achieved in ATR-FTIR was applied to the molecular structure characterisation to provide details on the explanation of the adhesion process. In Figure 2, the ATR-FTIR spectra are shown for TPU and PA6. As reported in the literature, the hard segments of TPU contain NH and C=O groups with characteristic vibrations that can interact and form intermolecular hydrogen bonds. Thus, TPU was characterised by a band in the region from 3000 cm^−1^ to 3500 cm^−1^ due to N-H stretching vibrations. The absorption band of the most polar group of TPU, namely the carbonyl group (C=O), is located in the wavenumber range from 1800 cm^−1^ to 1640 cm^−1^ and results from a hydrogen-bonded carbonyl stretching vibration [23,24]. The characteristic absorption bands of the ATR-FTIR spectra of PA6 are represented in Figure 2. At approximately 3300 cm^−1^, the band ascribed to the stretching vibration of the amine groups (N-H bending) was detected. At 2930 cm^−1,^ and 2855 cm^−1,^, the asymmetric and symmetric stretching vibration bands of the CH_2_ and CH_3_ groups are represented. The wavenumber positions at 1633 cm^−1^ and 1541 cm^−1^ of the amide I [v(C=O] and amide II [(N-H), (C-N)] bonds were recorded, respectively. The bands ascribed to C-H scissor deformation (CH_2_) and (C-H) symmetric bending in (CH_3_), were appearing at 1465 cm^−1^, and 1370 cm^−1^. At about 940 cm^−1^ and 685 cm^−1^, bands due to C–C=O stretching vibration and N–H out-of-plane bending vibration were measured, respectively [25].

#### 3.1.2. Decomposition Behaviour

**Figure 3:**
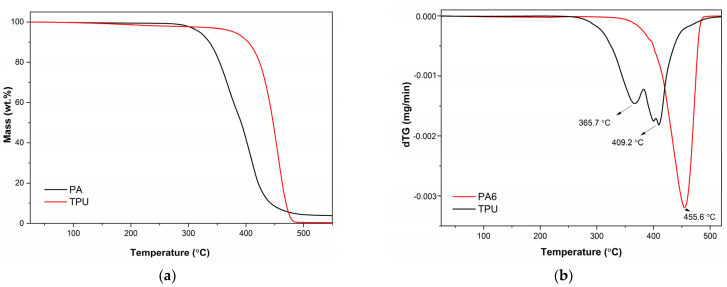
TGA measurements results of PA6 and TPU: (**a**) mass loss as a function of temperature; (**b**) dTG curves.

The thermal decomposition behaviour of both materials, PA6 and TPU, were studied with non-isothermal TGA measurements or with isothermal measurements. The non-isothermal measurements were evaluated over a temperature range of 40 °C to 550 °C. Figure 3 shows the TGA thermograms and derivative thermogravimetry (dTG) curves of the TPU and PA recorded at a heating rate of 10 K/min. The initial decomposition temperatures determined at the onset of the TGA thermograms were 335 °C and 424 °C for the TPU and PA6, respectively. The decomposition was carried out as a one-step process for the PA material and a two-step process for the TPU material. According to the figure, from the dTG curves, the highest decomposition rates were found to be at 366 °C and 409 °C for TPU, with the first decomposition step being ascribed to the cleavage of urethane bonds, while the second step was ascribed to aliphatic segments [26]. On the other hand, the PA6 decomposed in a single step with the highest decomposition rate at 456 °C. The residual mass after the pyrolytic decomposition for TPU was around 4%, while that for PA6 was almost completely decomposed. Overall, the non-isothermal TGA results suggest a significantly better thermal stability of PA6 in comparison to TPU.

**Figure 4:**
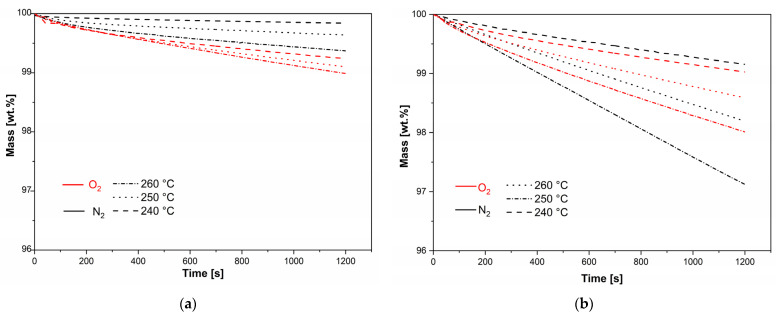
Isothermal measurements results: (**a**) PA6; (**b**) TPU.

The isothermal TGA measurements can provide additional insights into the processing window of polymeric materials [27,28,29]. Therefore, the thermal decomposition of TPU and PA6 was also characterised using isothermal TGA measurements in either a N_2_ or O_2_ atmosphere, with the results presented in Figure 4. The measurements were performed in the temperature range of 240 °C to 260 °C, which corresponds to the printing temperatures of TPU. As expected, both materials exhibited increasing rates of decomposition with increasing temperatures. The PA6 exhibited a relatively low mass loss at the investigated temperature range, with only about a 1% mass loss occurring at 260 °C, in an O_2_ atmosphere and with a time of 1200 s. The mass losses of PA6 were higher in the O_2_ atmosphere, independent of the temperature. On the other hand, TPU exhibited significantly higher mass losses compared to PA6, with about a 3% mass loss occurring at 260 °C, in N_2_ atmosphere and with a time of 1200 s. Moreover, in contrast to PA6, TPU exhibited higher decomposition rates in the N_2_ atmosphere at temperatures above 240 °C, which was ascribed to a partial compensation of mass loss due to oxidation reactions.

#### 3.1.3. Thermal Properties

**Figure 5:**
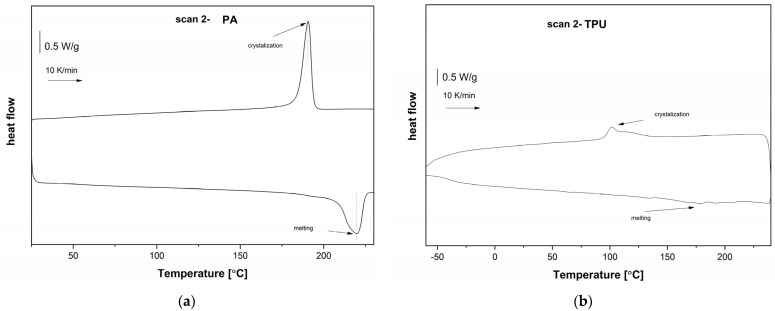
DSC thermograms of the first heating and second cooling runs of (**a**) PA6; (**b**) TPU.

DSC was employed to investigate structural differences following the glass transition, crystallisation and melting temperature of both materials, TPU and PA. Therefore, the first cooling run and second heating run were evaluated (after erasing the thermal history), with corresponding thermograms represented in Figure 5. Upon heating, both materials exhibited the main glass transition steps and melting endothermal peaks, which were complex in the case of TPU. The results were studied in terms of characteristic temperatures, namely, of the glass transition (*T*_g_) and melting (*T*_m_). Upon heating, TPU exhibited a *T*_g_ of −39 °C, obtained by the half-heat capacity change (Δ*c*_p_) of 0.25 J/gK, indicating a relatively high degree of amorphous morphology. TPU exhibited a broad melting range with a melting enthalpy (Δ*H*_m_) of 14 J/g and multiple endothermic peaks located at 135 °C, 178 °C, 192 °C and 204 °C. The complexity of the melting endotherm is termed as multiple melting points and is related to the disordering of crystallites corresponding to type I and type II with a relatively short-range order. Upon cooling, TPU exhibited a relatively broad exothermic crystallisation peak, with a crystallisation temperature (*T*_c_) of 102 °C and a crystallisation enthalpy (Δ*H*_c_) of 14 J/g [30]. Upon heating, PA6 exhibited a *T*_g_ of 51 °C, with Δ*c*_p_ equalling 0.03 J/gK, and an endothermic melting peak with a *T*_m_ of 220 °C and Δ*H*_m_ of 69 J/g, equalling a degree of crystallinity of 30%. Upon cooling, PA6 exhibited an exothermic crystallisation peak, with a *T*_c_ of 190 °C and Δ*H*_c_ of 69 J/g.

### 3.2. Substrate Characterisation

#### 3.2.1. Surface Topology

**Figure 6:**
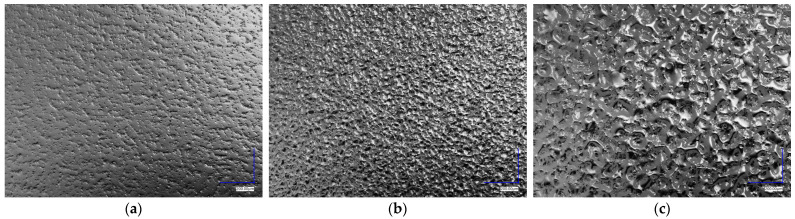
Optical micrographs of the substrate surfaces with targeted roughness of (**a**) 0.2 µm; (**b**) 0.8 µm; (**c**) 3.2 µm.

Although the targeted line roughness of the part with each tool insert is known, there are many influencing factors determining the final roughness of the part, with material viscosity and processing parameters being among the most important. Therefore, the substrate surfaces were investigated through optical microscopy and surface roughness measurements using a confocal microscope. Optical micrographs of the injection moulded substrate surfaces with varying targeted roughnesses and corresponding measured values are presented in Figure 6 and Table 4, respectively. The optical micrographs reveal significant differences between the topologies of the substrates, with the substrates injection moulded into more textured cavities having visibly higher roughnesses. This observation was further confirmed and quantified through surface roughness measurements using a confocal microscope. The measured values of both the *R*_a_ and *S*_a_ of substrates R6 and R18 corresponded well to the target values, while substrate R30 exhibited higher values of *R*_a_ and *S*_a_ by approximately 1.8 µm and 1.3 µm, respectively.

#### 3.2.2. Wettability

**Figure 7:**
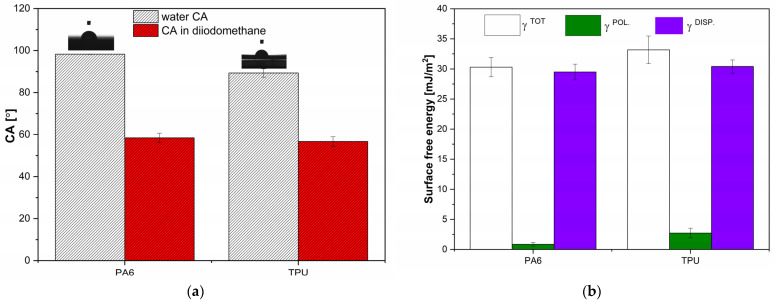
(**a**) Static water contact angles on PA6 and TPU with (**b**) corresponding surface free energy calculations of PA6 and TPU using Owens and Wend models.

The good wettability of a polymer surface is essential for providing a high interfacial strength. The wettability of a polymer surface is related to the interplay between the adhesive forces between the solid surface and the liquid and cohesive forces between the molecules in the liquid. Furthermore, wettability is influenced not only by the surface free energy that depends on the chemical composition and supermolecular structure but also, by the surface topology [31]. As can be seen from Figure 7, the wettability of each material varies. The water contact angle of both PA6 and TPU reflect hydrophobic surfaces due to the average contact angle of up to 100°. The water contact angle of PA6 was close to 98° and that of TPU was 89°. On the other hand, the contact angles between the polymer surfaces were similar, with values of 58° and 56° for PA6 and TPU, respectively. Owens and Wendt models were used to interpret the surface free energy of PA6 and TPU. The γ_s (tot)_ of PA6 was about 30.3 mJ/m^2^ with a small polar fraction (0.86 mJ/m^2^) and a large disperse fraction (29.5 mJ/m^2^). The γ_s (tot)_ of TPU was higher, at about 33.1 mJ/m^2^ due to both a higher polar fraction (2.7 mJ/m^2^) and disperse fraction (30.4 mJ/m^2^). However, while the disperse fractions of both materials are relatively similar, there is a large difference in their polar fraction, indicating that the surface treatments of PA6 that increase the polar fraction of surface free energy may result in better wettability and higher adhesion and, in turn, higher bonding strength.

### 3.3. Characterisation of the Bonding Strength

#### 3.3.1. The Influences of Interfacial Area and Substrate Roughness

**Figure 8:**
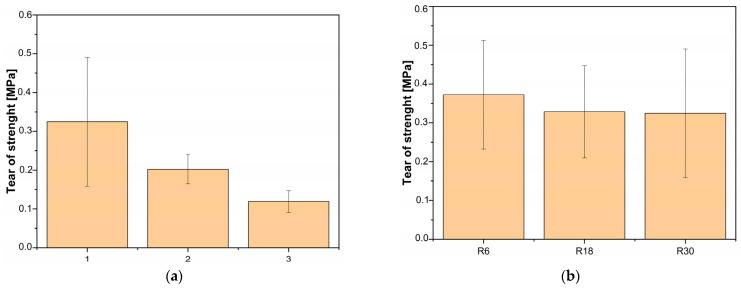
The tear-off strength dependence on the (**a**) interfacial area; (**b**) substrate roughness.

The influences of the interfacial area and substrate roughness on the value of tear-off strength are presented in Figure 8a and Figure 8b, respectively. While there were no statistically significant differences (α = 0.05) between the tear-off strength of the rib with thicknesses of 9 mm and 6 mm, the general trend is that tear-off strength decreases with increasing interfacial area between the substrate and the rib. The average values of tear-off strength decreased from around 0.3 MPa for a thickness of 3 mm to around 0.2 MPa and 0.1 MPa for thicknesses of 6 mm and 9 mm, respectively. The lower values of tear-off strength at samples with higher interfacial areas were ascribed to greater residual stresses resulting from non-uniform cooling rates due to the temperature gradient. With each deposited layer, the residual stresses in the part increase and can lead to the peeling of the part if the adhesion is not sufficient [32]. Similarly, a higher material volume in the part leads to a higher overall shrinkage and, consequently, higher residual stresses and, in turn, a lower tear-off strength. On the other hand, the influence of the surface roughness on the tear-off strength was not significant. Samples overprinted at substrates with varied surface roughnesses had tear-off strengths in a range of around 0.3 MPa to 0.4 MPa, with the highest value associated with the sample with the lowest surface roughness. Higher surface roughness should lead to better adhesion between the polymer surfaces due to a higher specific surface area and additional mechanical interlocking, which was not the case in our study [33]. Possible explanations for this are that the material viscosity being too high to effectively fill the pits of the rougher surface, the peaks melting due to heat transfer from the extruder material or the effect being so small that it was not visible due to high deviations in the measured values.

#### 3.3.2. The Influences of the Processing Parameters

An analysis of variance (ANOVA) was performed to determine factors with statistically significant influence on the tear-off strength. A significance level (α) of 0.05 was considered in the analysis, and all non-significant (*p*-value > 0.05) model terms were excluded from the model. The ANOVA results are presented in Table 5. All three main factors were shown to be statistically significant, with a relatively low probability of Type I error (below 2%). The only significant non-linear response was found for printing speed, while significance was found only for interactions between printing and the substrate temperatures.

**Figure 9:**
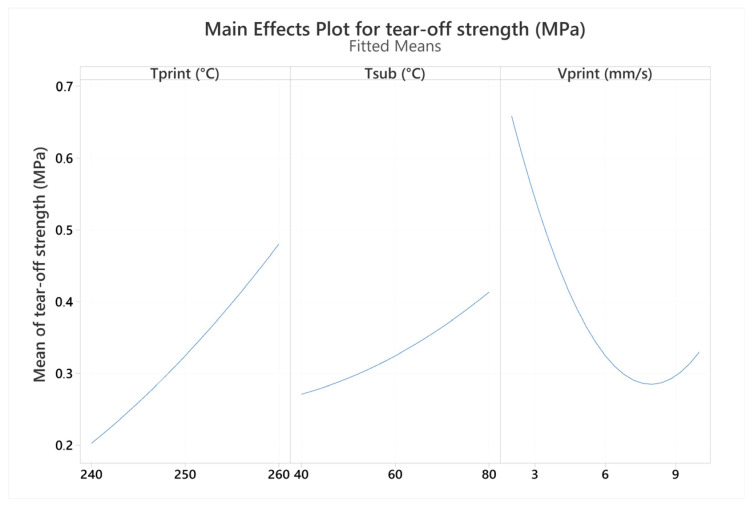
Main effect plot for tear-off strength.

**Figure 10:**
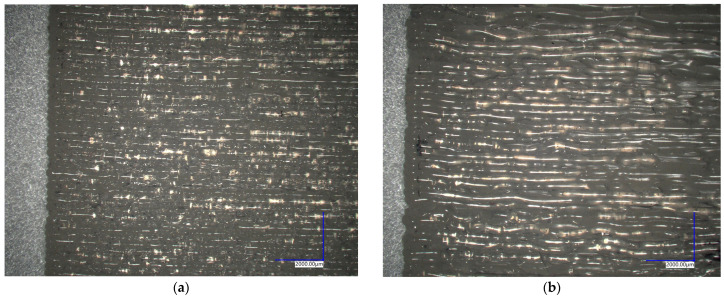
Optical micrographs of ribs 3D printed at low and high printing temperatures: (**a**) sample 240-60-2; (**b**) sample 260-60-2.

The main effect plot for tear-off strength is presented in Figure 9. Increasing both printing and substrate temperatures resulted in an increased tear-off strength with a linear response, although with different magnitudes of effects. It was shown that on average, the printing temperature has a larger influence compared to the substrate temperature in the investigated temperature range. While further increases in printing and substrate temperatures may result in further increases in tear-off strength, they could also result in some detrimental effects. The TGA results revealed that TPU exhibited noticeable degradation when exposed to a temperature of 240 °C for a relatively short period, with progressively higher degradation rates with further increases in temperatures. Moreover, optical micrographs of ribs produced at low and high printing temperatures, as presented in Figure 10, revealed that the layers are unevenly stacked, and the extruded strands have wavy structures in the case of a high (260 °C) printing temperature, while samples printed at 240 °C have more uniformly deposited layers with straighter strands. The uneven stacking of layers in the sample printed at high temperature may arise due to a too low viscosity of the extruded material and postponed solidification that results in the unwanted deformation of extruded strands. In addition, the sample printed at 260 °C had visible bubbles in the layers that were ascribed to the material decomposition and associated formation of decomposition gases, while there were no bubbles visible in the sample printed at 240 °C. Printing speed had the largest effect on tear-off strength among the investigated factors, with a distinctly nonlinear response. High and middle values of printing speed resulted in approximately the same values of tear-off strength, while at low values of printing speed, the tear-off strength was significantly higher. Low printing speeds may result in a heat transfer from a nozzle to the substrate that is sufficient for the effective melting of the surface, which enables better bonding.

**Figure 11:**
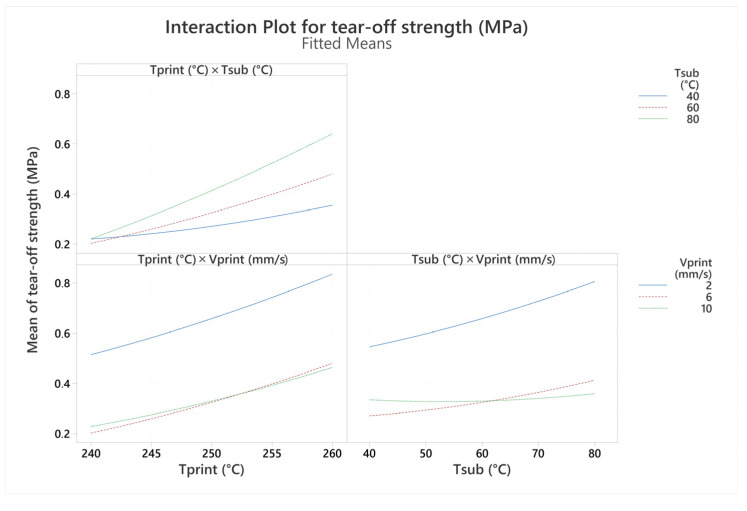
Interaction plots for tear-off strength.

The interaction plot for tear-off strength is presented in Figure 11. The only term that statistically significantly influenced the tear-off strength is the interaction between the printing and substrate temperatures. At low printing temperatures, the values of tear-off strength are relatively low (around 0.2 MPa), independent of the substrate temperature. With increasing printing temperatures, the bed temperature had a progressively higher effect on the tear-off strength, with higher substrate temperatures resulting in higher tear-off strengths. This effect may be due to insufficient heat transfer from the extruded material at low printing temperatures to allow for the sufficient melting of the substrate so that the polymers can melt together. In contrast, at higher printing temperatures where the surface can be melted, the higher substrate temperatures facilitate the intermolecular diffusion and entanglement of polymer chains, leading to increased adhesion [7].

## 4. Conclusions

The bonding behaviour between injection moulded PA6 parts and overprinted TPU components was studied. PA6 was injection moulded into a mould with cavities with different textures to obtain substrates with varying surface roughnesses. Rib structures were printed onto substrates using a desktop FFF printer using commercially available TPU filament by varying the interfacial area between the components, substrate surface roughness and processing parameters (printing temperature, substrate temperature and printing speed). The chemical composition and thermal properties as well as surface properties of the prepared specimens were studied to gain a deeper insight into adhesion phenomena including chemical bonding and interaction, diffusion and mechanical interlocking. It was found that TPU has a relatively poor thermal stability, which combined with a high melting temperature, results in a narrow processing window. Surface-free energy calculations revealed that TPU has higher surface free energy, especially due to the polar fraction. It was found that tear-off strength decreases with an increased interfacial area, which was ascribed to higher residual stresses in the overprinted structure, resulting from shrinkage. In contrast, it was shown that substrate surface roughness does not significantly influence the tear-off strength, despite a theoretically greater potential for mechanical interlocking. Printing speed has the largest influence on the tear-off strength among the investigated processing parameters, with the lowest factor level leading to the highest tear-off strength. Increasing both the printing and substrate temperature led to higher values in tear-off strength, with the former factor having an effect of higher magnitude. In addition, it was also clearly determined that high substrate temperatures result in a significant increase in tear-off strength only at high printing temperatures. Overall, the results indicate that TPU can be successfully overprinted onto PA6 injection moulded parts. However, the bonding strength between the components is relatively low compared to the typical bonding strengths between the injection over-moulded components. Based on the results, it can be concluded that to achieve optimal bonding strength, the printing speed of the first layer should be kept at a low value, while both the printing temperature and substrate temperature should be maximised to the point that allows the production of parts without defects. In addition to the processing parameters, the overprinted part geometry seems to play a very important role, with thinner structures resulting in better bonding strengths due to less residual stresses. Two main strategies seem promising to further increase the bonding strength between the PA6 injection moulded parts and TPU: a) the surface treatment strategies that increase the polarity of the PA6 surface may contribute to better wettability and higher adhesive forces and b) the modification of TPU by blending it with fillers that decrease the shrinkage may result in lower residual stresses in the overprinted structures.

## Figures and Tables

**Table 1:** Processing parameters for the injection moulding of the test specimens.

Processing Parameter	Values PA6	Valus PA6—Substrates	Values TPU
Temperature profile (nozzle to hopper) (°C)	230, 235, 230, 225, 220	230, 235, 230, 225, 220	200, 195, 190, 185, 180
Mould temperature (°C)	80	80	40
Metering stroke (mm)	18	50	19
Decompression (mm)	5	2	2
Screw angular velocity (min^−1^)	50	50	80
Backpressure (MPa)	2.5	2.5	2.5
Injection velocity (mm/s)	50	20	50
Switch-over point (mm)	10	20	10
Packing pressure (MPa)	30	30	50
Packing time (s)	10	10	10
Rest cooling time (s)	20	20	15

**Table 2:** Processing parameters used for the overprinting of samples with varying interfacial areas and substrate surface roughnesses.

Processing Parameter	Values and Units
Nozzle diameter	0.4 mm
Layer height	0.28 mm
Infill density	100%
Infill pattern	Lines
Line width	0.4 mm
Wall thickness	0.8 mm
Cooling fan	Disabled
Print speed of subsequent layers	20 mm/s
Number of initial layers	2
Printing temperature (*T*_print_)	250 °C
Substrate temperature (*T*_sub_)	60 °C
Initial layer speed (*V*_print_)	6 mm/s

**Table 3:** The Box–Behnken experimental design for the overprinting of ribs onto substrates.

Sample	Standard Order	Run Order	*T_print_*	*T*_substrate_	*V_print_*
240-40-6	1	1	240	40	6
260-40-6	2	10	260	40	6
240-80-6	3	6	240	80	6
260-80-6	4	5	260	80	6
240-60-2	5	7	240	60	2
260-60-2	6	11	260	60	2
240-60-10	7	12	240	60	10
260-70-10	8	8	260	70	10
250-40-2	9	9	250	40	2
250-80-2	10	4	250	80	2
250-40-10	11	3	250	40	10
250-80-10	12	13	250	80	10
250-60-6	13	2	250	60	6

**Table 4:** The measured values of the surface roughnesses of the substrates injection moulded into cavities with different textures.

Substrate	Targeted Roughness (µm)	*R*_a_ (µm)	S_a_ (µm)
R6	0.2	0.17 ± 0.03	0.19
R18	0.8	0.84 ± 0.04	0.94
R30	3.2	4.96 ± 0.24	4.52

**Table 5:** ANOVA results of the response surface regression.

Source	DF	Adj SS	Adj MS	F-Value	*p*-Value
Model	9	0.525654	0.058406	31.43	0.008
Linear	3	0.410889	0.136963	73.71	0.003
*T*_print_ (°C)	1	0.154196	0.154196	82.98	0.003
*T*_sub_ (°C)	1	0.040477	0.040477	21.78	0.019
*V*_print_ (mm/s)	1	0.216216	0.216216	116.36	0.002
Square	3	0.078828	0.026276	14.14	0.028
*T*_print_ (°C) × *T*_print_ (°C)	1	0.000643	0.000643	0.35	0.598
*T*_sub_ (°C) × *T*_sub_ (°C)	1	0.000716	0.000716	0.39	0.579
*V*_print_ (mm/s) × *V*_print_ (mm/s)	1	0.065618	0.065618	35.31	0.010
2-Way Interaction	3	0.035937	0.011979	6.45	0.080
*T*_print_ (°C) × *T*_sub_ (°C)	1	0.020289	0.020289	10.92	0.046
*T*_print_ (°C) × *V*_print_ (mm/s)	1	0.001828	0.001828	0.98	0.394
*T*_sub_ (°C) × *V*_print_ (mm/s)	1	0.013819	0.013819	7.44	0.072
Error	3	0.005574	0.001858		
Total	12	0.531228			

## Data Availability

Data are contained within the article.
